# Prevalence of Pelvic Floor Dysfunction in Women After Pelvic Radiotherapy: Systematic Review and Meta-Analysis

**DOI:** 10.1007/s00192-026-06557-z

**Published:** 2026-03-13

**Authors:** Constanza Deyanu Alvear Pérez, Anna Karoline Lopes Rocha, Maria Palharini Volpato, Cassia Raquel Teatin Juliato, Simone Botelho Pereira, Cassio Luis Zanettini Riccetto

**Affiliations:** 1 Department of Surgery, Postgraduate Program in Surgery Sciences, School of Medical Sciences, University of Campinas (UNICAMP), Rua Tessália Vieira de Camargo, 126, Cidade Universitária “Zeferino Vaz”, Campinas, SP 13083-887 Brazil; 2 Department of Obstetrics and Gynecology, School of Medical Sciences, University of Campinas (UNICAMP), Campinas, SP Brazil; 3 Motor Science Institute, Postgraduate Program in Rehabilitation Sciences, Federal University of Alfenas (UNIFAL-MG), Alfenas, Minas Gerais Brazil; 4 Division of Female Urology, School of Medical Sciences, University of Campinas (UNICAMP), Campinas, SP Brazil

**Keywords:** Adjuvant, Chemoradiotherapy, Pelvic Floor Disorders, Pelvis, Radiotherapy

## Abstract

**Introduction and Hypothesis:**

Survivors of pelvic cancer are at risk for pelvic floor dysfunction (PFD). This review is aimed at determining the pooled prevalence of PFD after pelvic radiotherapy and explore associations with treatment modalities.

**Methods:**

We included observational studies of women with urogynecological or anorectal cancer treated with radiotherapy, brachytherapy, or chemoradiotherapy, assessing PFD. Exclusion criteria were previous pelvic surgery or rehabilitation, reviews, case reports, conference abstracts, comments, letters, or inaccessible full texts. We searched PubMed, PubMed PMC, Cochrane library, EMBASE, Web of Science, and Scopus to 6 August 2024. Risk of bias was assessed using the Joanna Briggs Institute Critical Appraisal Checklist for Prevalence Studies. Meta-analyses were conducted in R software. Meta-regression explored potential covariates. A 5% level of statistical significance was established.

**Results:**

Fifteen studies involving 1667 women were included. Pooled prevalence estimates were urinary incontinence (UI, 37%), stress UI (34%) and urgency UI (47%), overactive bladder (42%), anal incontinence (24%), defecatory urgency (20%), pelvic pain (30%), and sexual dysfunction (19%). Longer follow-up time was associated with increased defecatory urgency (*p* = 0.0054). Certainty of evidence was moderate for urgency urinary incontinence and overactive bladder, and low for the other outcomes. No publication bias was detected (Egger’s test, *p* = 0.17).

**Conclusions:**

Radiotherapy for pelvic malignancies is associated with UI and overactive bladder symptoms. Bowel and sexual dysfunction were heterogeneous, but defecatory urgency increased over time. Limitations include heterogeneity in follow-up and study methods. Routine assessment and proactive multidisciplinary care are needed to mitigate morbidity and improve quality of life.

**Supplementary Information:**

The online version contains supplementary material available at https://doi.org/10.1007/s00192-026-06557-z

## Introduction

Pelvic malignancies in women represent a major global health concern. Colorectal cancer is the third most common cancer overall, and the second most frequent among women in countries undergoing epidemiological transition, with an incidence of 20 per 100,000 compared with 18.8 per 100,000 for cervical cancer.

Cervical cancer is the fourth most common cancer worldwide and the second most frequent among women, with an estimated 604,000 new cases in 2020. Endometrial cancer ranks sixth globally, with approximately 417,000 new cases, and is the most common gynecological malignancy in high-income countries [1].

Radiotherapy (RT)—delivered as external beam radiation therapy, brachytherapy, or chemoradiotherapy—plays a central role in the management of advanced pelvic malignancies when surgery is not a viable option. Although effective in achieving local tumor control and reducing recurrence rates, radiotherapy inevitably exposes surrounding structures, including pelvic organs, musculature, and connective tissues, to radiation damage [2]. Consequences are particularly relevant in the gastrointestinal, genitourinary, and musculoskeletal systems of the pelvic floor.

The pelvic floor (PF) is a highly integrated anatomical and functional unit that provides support to pelvic organs while ensuring urinary, anorectal, and sexual function [3]. Radiation-induced injury can disrupt these mechanisms, leading to pelvic floor dysfunction (PFD) such as urinary incontinence (UI), overactive bladder, anal incontinence, and sexual dysfunction [4]. These conditions may substantially impair quality of life and daily functioning [5, 6].

Advances in screening and treatment have improved survival among women with pelvic cancers [4], but survivorship also raises new challenges. The prevalence and profile of PFD after radiotherapy remain insufficiently described, despite their clinical importance.

A clearer understanding of their frequency is essential for designing evidence-based follow-up care strategies. If prevalence is higher than currently anticipated, or if certain dysfunctions are underrecognized, clinical algorithms may need adjustment to better address long-term morbidity.

Existing reviews are limited. Shan et al. (2023) provided meta-analyses focused on cervical cancer survivors, but their scope was restricted to selected symptoms and included women who had also undergone surgery [7]. Bernard et al. (2016) examined the functional effects of pelvic radiotherapy on pelvic floor muscles but did not assess the prevalence of PFD [8]. To our knowledge, no prior review has quantified the prevalence of all PFD specifically in women treated with pelvic radiotherapy without surgery. Our study therefore fills this gap by offering comprehensive prevalence estimates across pelvic radiotherapy-based treatment.

This review is aimed at determining the prevalence of PFD in women following pelvic RT. Specifically, we seek to describe urinary, defecatory, and sexual dysfunction and to explore their associations with age and time since treatment. Accordingly, the review addresses the following research questions: what are the prevalence and profile of PFD among women after pelvic radiotherapy, and how is this dysfunction related to patient age and time elapsed since treatment?

## Materials and Methods

This systematic review was conducted according to the Preferred Reporting Items for Systematic Reviews and Meta-Analyses Statement (Supplementary material 1) [9]. Ethical approval was not required by the Institutional Review Board as the review did not involve primary data collection. However, the review protocol was registered at PROSPERO (CRD420251054275).

### Search Strategy and Selection Criteria

We systematically searched PubMed, PubMed PMC, BVS, Scorpus, Web of Science, Embase, and The Cochrane Library up to 6 August 2024. Reference lists of eligible articles were also screened. The following search terms were applied: (Women OR Female) AND (Radiotherapy OR Chemoradiotherapy OR Radiotherapy, Adjuvant OR Brachytherapy) AND (Pelvic Floor Disorders OR Pelvis OR Pelvic Floor). Details are given in Supplementary material 1.

We also included additional sources identified through the reference lists of excluded systematic reviews. Technical support for the search was provided by the Library from State University of Campinas (UNICAMP), Campinas, SP, Brazil. Two reviewers (C.A., A.R.) independently screened titles, abstracts, and full texts using Rayyan software. Discrepancies were resolved by consensus with a third reviewer (M.V.). The studies selected are described in Table 1.

**Table 1:** Characteristics of the studies included

Reference	*N*	Age (mean ± SD/range)	Tumor stage				Radiation dose (Gy)	BT	CT	Months of follow-up (median ± SD/range)
			I	II	III	IV				
Cui et al. [6]	120	29–76	16	41	57	6		43	102	
Hazewinkel et al. [10]	73	28–92		42		58			67	
Zhu et al. [11]	31	56.39 ± 13.83		2	6	23			29	
Miguel et al. [4]	32	47 ± 11, 28–65	2	20	10		45	32	32	6–9
Abayomi et al. [12]	95	62 ± 14.2, 28–90		36		10	45–50.4	78	26	3–336
Dunberger et al. [13]	61	64.4, 28–79					< 38 and 54 (SD 7.8, 39.6—70)	50	32	86.1 ± 40.4^a^
Hazewinkel et al. [14]	47	52 ± 12	34			66			4	72, 12–132
Kirchheiner et al. [15]	588	49, 22–91	105	332	114	23	45—50	588	545	36–60
Lind et al. [16]	67	28–79						52	32	74, 26–179
Parkin et al. [17]	66	58.6 ± 11.3, 39–74	20	27	9		16.5 and 42.5	66		60–132
Vistad et al. [18]	91	27–82	13	65	12	1	45–60	91		100, 66–131
Georg et al. [19]	225	58, 26–92	25	137	53	10	45–50.4	225	147	44, 3–84
Hsu et al. [3]	111	66.62 ± 8.92, 42–80		111			40–46			46, 25–86
Noronha et al. [20]	40	52.9 ± 7.2	5	12	22	1			20	
Zomkowski et al. [21]	20	18–70	3	16	1					

*BT* brachytherapy, *CT* chemotherapy

^a^Follow-up with mean and SD

#### Inclusion Criteria

The inclusion criteria were women diagnosed with urogynecology or anorectal cancer, regardless of stage; treatment with radiotherapy, brachytherapy or chemoradiotherapy; observational study design (cross-sectional, cohort, or prospective); assessment of PFD after treatment.

#### Exclusion Criteria

The exclusion criteria were previous pelvic surgery or rehabilitation treatment; reviews; case reports; conference abstracts; comments or letters; non-accessible full texts.

### Data Extraction

Data extraction was performed independently by two reviewers. Extracted variables included: author, year of publication, study design, sample size and age, cancer type, treatment modality, time of evaluation, number of women with PFD, and specific dysfunction reported. Articles lacking certain descriptive variables were still retained if they met the inclusion criteria. Disagreements were resolved by consensus with the third reviewer. The types of dysfunction identified were grouped by type for the meta-analysis (Table 2).

**Table 2:** Prevalence of pelvic floor dysfunction after pelvic radiotherapy

Variables	Number of studies	*N*	Prevalence (%)	CI (%)	I^2^ (%)	*p* value
Urinary dysfunction						
Urinary incontinence	9	805	37	27–47	88.4	< 0.0001
Stress urinary incontinence	5	305	34	25–45	68.8	0.0122
Urgency urinary incontinence	5	305	47	40–53	18.6	0.2961
Overactive bladder	6	349	42	35–50	46.4	0.0965
Increased urinary frequency	7	700	27	12–50	94	< 0.0001
Bowel dysfunction						
Anal incontinence	8	684	24	11–44	88.6	< 0.0001
Liquid stool incontinence	4	270	32	18–50	86	< 0.0001
Defecatory urgency	8	658	20	10–38	93.4	< 0.0001
Constipation	4	308	15	10–23	45.1	0.1405
Pain and sexual dysfunction						
Pelvic pain	7	490	30	19–43	87.6	< 0.0001
Sexual dysfunction	5	877	19	14–25	62.9	0.0293
Dyspareunia	4	857	9	6–15	76	0.0059
Vaginal dryness	4	857	10	4–21	91.4	0.0973

*p* value: according to the test of heterogeneity between studies

### Methodological Quality Assessment

**Fig. 1:**
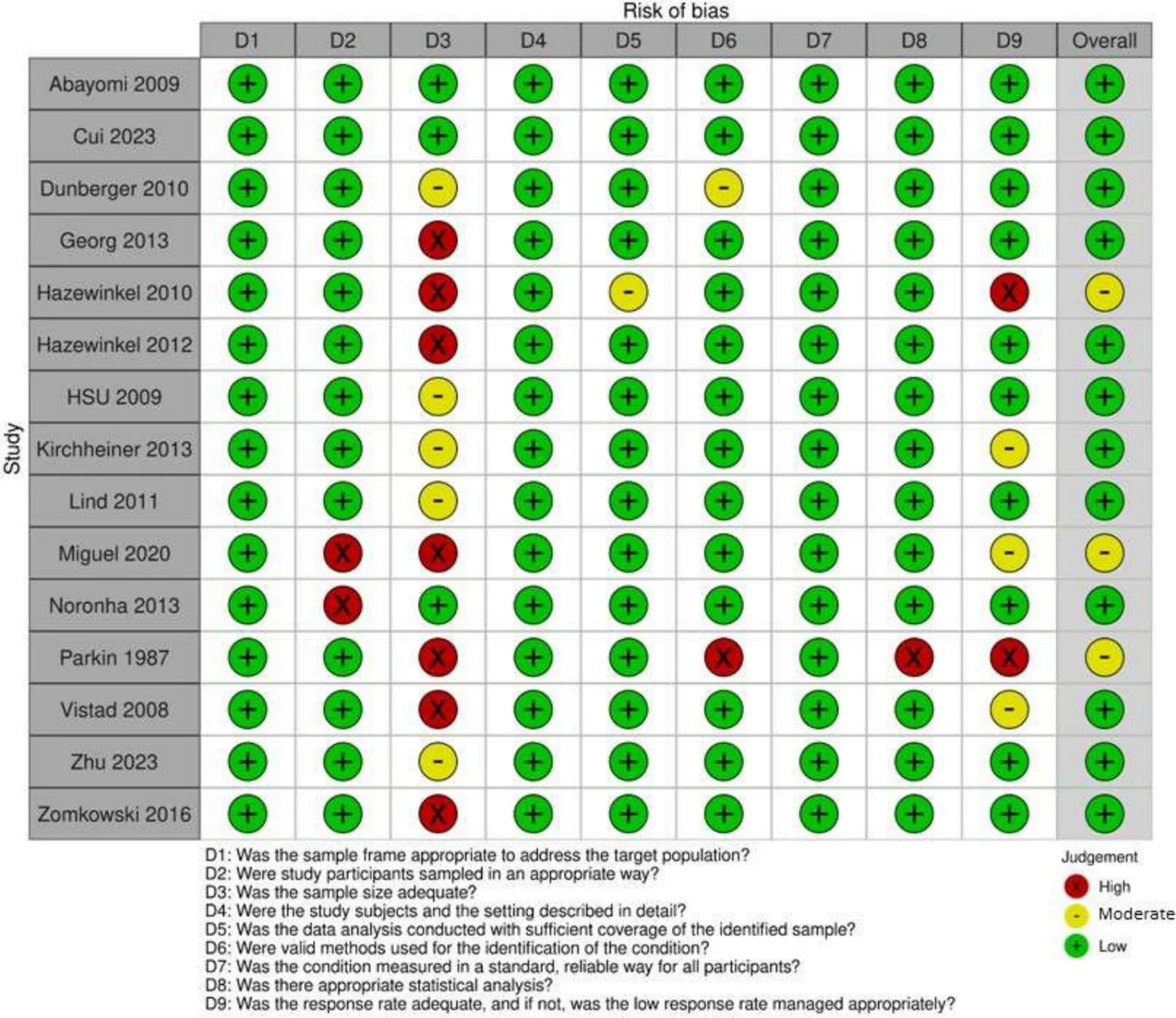
Joanna Briggs Institute checklist: risk

Risk of bias was assessed using the Joanna Briggs Institute (JBI) Critical Appraisal Checklist for Prevalence Studies. Two reviewers independently rated the nine items (yes, no, unclear or not applicable; Fig. 1) [22]. Discrepancies were settled by discussion and agreement with a third reviewer.

### Statistical Analysis

Pooled prevalence estimates and heterogeneity were obtained through meta-analyses using the inverse variance method, with between-study variance estimated by restricted maximum likelihood. Confidence intervals for variance and confidence intervals were obtained using the Q-profile method. Heterogeneity was assessed using Cochran’s Q test and quantified with I^2^.

Proportions were stabilized using logit transformation. Meta-regression analyses were conducted to explore the effect of potential covariates on the pooled estimates. This method models the relationship between study-level characteristics (moderators) and the observed effect sizes, using summary-level data. In addition, residual heterogeneity was assessed.

To ensure the robustness of pooled results we checked all references in order to remove studies with a high risk of bias.

To explore potential publication bias, funneling asymmetry was visually inspected, and Egger’s linear regression test was applied to statistically assess asymmetry.

All analyses were performed using R software (R Core Team, 2024), using the meta and metafor packages. The level of statistical significance was *p* < 0.05.

## Results

### Study Selection

**Fig. 2:**
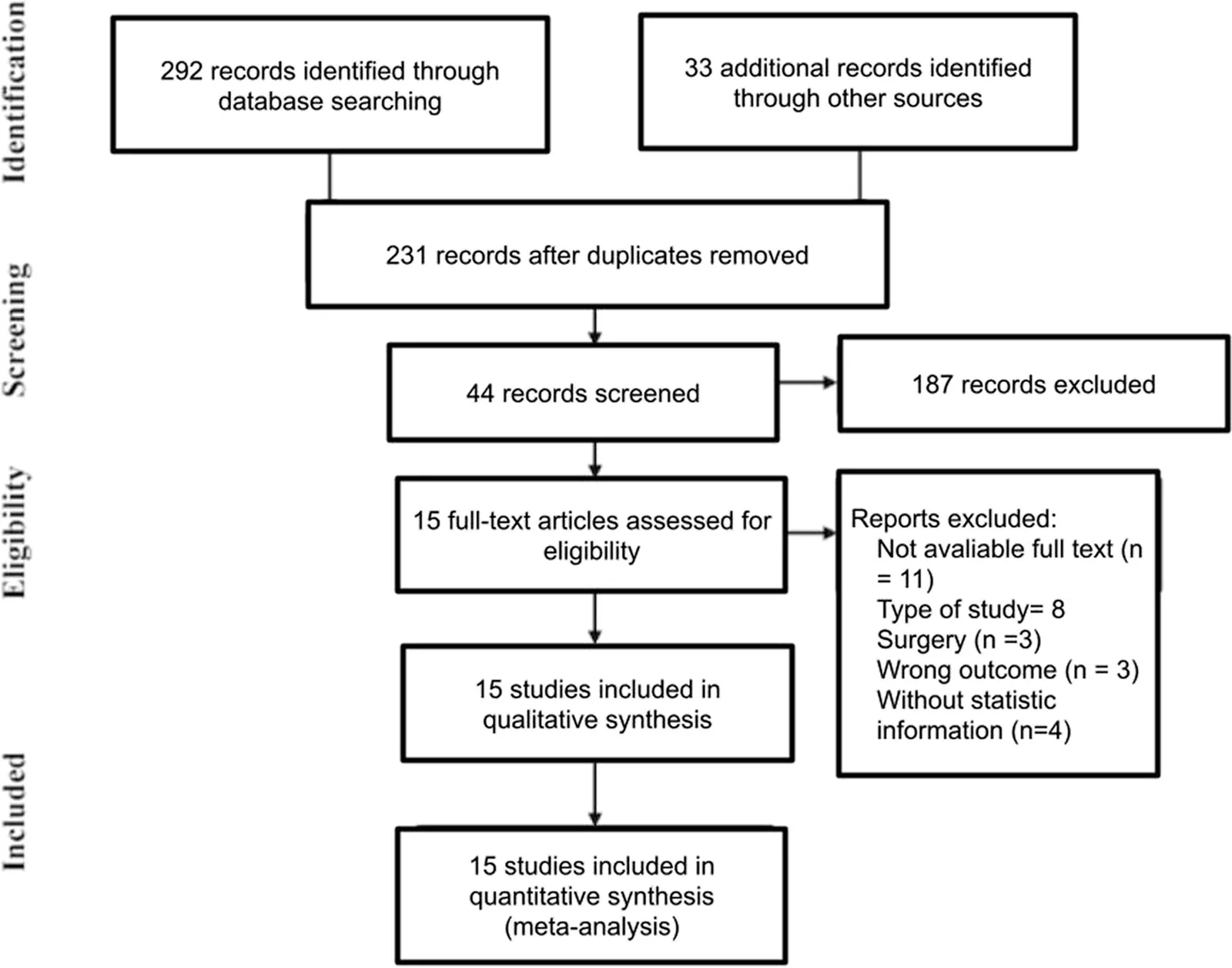
Preferred Reporting Items for Systematic Reviews and Meta-Analyses flowchart of screening and selection phases

Of 325 studies initially identified, 15 met the eligibility criteria, with a total of 1667 participants included in the meta-analysis (Fig. 2, Table 1).

### Prevalence of PFD

#### Urinary Dysfunction

Nine studies (*n* = 805) reported urinary incontinence with a pooled prevalence of 37% (95% CI 27–47%), high heterogeneity (I^2^ = 88.4%, *p* < 0.0001).

Stress urinary incontinence (SUI; five studies, *n* = 305) showed 34% (25–45%), moderate heterogeneity (I^2^ = 68.8%, *p* = 0.0122). Urgency urinary incontinence (UUI; five studies, *n* = 305) showed 47% (40–53%), no heterogeneous studies (I^2^ = 18.6%, *p* = 0.2961). Overactive bladder (six studies, *n* = 349), was 42% (35–50%) and homogeneous studies (I^2^ = 46.4%, *p* = 0.0965). Increased urinary frequency (seven studies, *n* = 700) reached 27% (12–50%), heterogeneity (I^2^ = 94%, *p* < 0.0001).

#### Bowel Symptoms

Anal incontinence (AI; eight studies, *n* = 684) showed 24% (11–44%), heterogeneity I^2^ = 88.6%, *p* < 0.0001. Liquid stool incontinence (four studies, *n* = 270) was 32% (18–50%), with heterogeneity I^2^ = 86%, *p* < 0.0001. Defecatory urgency (eight studies, *n* = 658) was 20% (10–38%), heterogeneity I^2^ = 93.4%, *p* < 0.0001. Constipation (four studies, *n* = 308) was 15% (10–23%) with homogeneous studies I^2^ = 45.1%, *p* = 0.1405.

#### Pain and Sexual Dysfunction

Pelvic pain (seven studies, *n* = 490), was 30% (19–43%), heterogeneous I^2^ = 87.6%, *p* < 0.0001. Sexual dysfunction (five studies, n = 877), was 19% (14–25%), heterogeneous I^2^ = 62.9%, *p* = 0.0293). Dyspareunia (four studies, *n* = 857) was 9% (6–15%) and heterogeneous studies I^2^ = 76.0%, *p* = 0.0059. Vaginal dryness (four studies, *n* = 857) was 10% (4–21%) with heterogeneity I^2^ = 91.4%, *p* = 0.0973.

Results are summarized in Table 2.

### Meta-Regression Findings

#### Urinary Incontinence

Mean age was not associated (*p* = 0.2169; I^2^ = 85.84%). Stage II disease was associated with lower risk (*p* = 0.0012).

#### Anal Incontinence

Chemotherapy was inversely associated with anal incontinence (*p* = 0.0116).

#### Defecatory Urgency

Chemotherapy was inversely associated (*p* = 0.0016). Longer follow-up time was positively associated with defecatory urgency (*p* = 0.0054). No consistent associations were found for overactive bladder, pelvic pain, or sexual dysfunction.

### Publication Bias

Funnel plot inspection and Egger’s test indicated no evidence of publication bias (*p* = 0.17).

### Study Quality

According to JBI criteria, risk of bias was rated as moderate in three studies and low in the remaining 12 (Fig. 1). Based on JBI, certainty of evidence varied by outcome as moderate for UUI and overactive bladder (OAB), and low for UI, AI, sexual dysfunction, dyspareunia, vaginal dryness, and pelvic pain.

### Sensitivity Analyses

As all the studies included were rated as having a low or moderate risk of bias, no exclusions were required, indicating the robustness of the pooled estimations (Fig. 1).

## Discussion

### Urinary Outcomes

The prevalence of UI across the studies was 37%, with 34% SUI, and 47% UUI. These values are higher than the global prevalence of UI (27.4%), where SUI is usually more common than UUI (20.1% vs 11.4% respectively) [23]. In contrast, our findings indicate a predominance of UUI over SUI in this population. OAB was reported in 42% of women, and increased urinary frequency in 27%, both markedly higher than the global prevalence of OAB (21.9%) [24]. These increased rates are consistent with the morphological and functional changes of the bladder after radiation exposure. In particular, urgency symptoms may be explained by fibrosis of the bladder wall, a decrease in compliance, and neuropathies [2, 25].

Age was not associated with UI in our analysis. Although aging is a recognized contributor to UI, owing to urethral tissue atrophy and reduced elasticity in menopause [26], radiotherapy itself appears to be a stronger determinant, impairing urethral coaptation through fibrosis and decreased wall elasticity [27]. Stage II disease was associated with a lower risk of UI. A possible explanation is that in larger tumors, besides the direct effect of radiation on the bladder wall, its spread may also compromise the bladder’s autonomic plexus, leading to an increase in afferent signals, thereby predisposing to more severe UI in advanced tumors. Symptom onset also appears to be time dependent. Georg et al. (2013) reported a mean onset of urinary symptoms at 27 ± 21 months (range 3–94 months), with 76% of cases appearing within 36 months after radiotherapy [19]. Owing to the limited number of studies, we were unable to perform meta-regression on follow-up time to verify this hypothesis.

### Bowel Outcomes

Anal incontinence was reported in 24% of patients, with liquid stool incontinence reaching 32%. Radiation can induce diarrhea owing to mucosal atrophy and enteritis [28]. Radiation can also cause or enhance previously occult striated anal sphincter deficiency. The levator anus complex is related mainly to continence for solid stools whereas the striate anal sphincter is mainly responsible for liquid stool continence [29]. Defecatory urgency (20%) and constipation (15%) may also result from fibrosis, reduced rectal distension volumes [30], and anal canal stenosis [28]. Our findings suggest an inverse relationship between chemotherapy and both anal incontinence and defecatory urgency. We propose that this finding is likely due to the strong negative impact of radiotherapy on these conditions, rather than chemotherapy itself having a protective effect. In our meta-regression a longer follow-up was positively associated with defecatory urgency, supporting the notion that symptoms can develop or worsen progressively after treatment. Indeed, in one of the studies included in our review, it was reported that rectal symptoms are often delayed (mean onset of 14 ± 8.5 months; range 3–34 months), with a highly variable duration of 19 ± 17 months (range 1–75 months). Regarding resolution, 81% of patients were complication free within 3 years, although it was unclear whether recovery was spontaneous or treatment related. In contrast, the lack of complete resolution in this timeframe of follow-up suggests a possible evolution toward chronicity [19].

### Sexual Dysfunction

Sexual dysfunction is another late effect of radiotherapy. In our analysis, 19% of women reported sexual dysfunction, with dyspareunia (9%) and vaginal dryness (10%) as more related complaints. Beyond physical alterations, psychological factors contribute substantially. Women may report diminished femininity, attractiveness, and confidence, as well as distress linked to vaginal symptoms (pain, dryness, shortening, loss of elasticity, and bleeding), all of which may lead to fear of sexual penetration and decreased sexual satisfaction [31]. Vaginal stenosis, which is progressive and persistent over time, reflects chronic fibroconnective tissue changes [15]. Although follow-up interval could not be analyzed owing to limited data, these symptoms are consistent with tissue remodeling, which compromises their sexual response.

### Pelvic Pain

Pelvic pain was present in 30% of patients in our review. Its etiology is multifactorial and may include peripheral neuropathy, fractures, enteritis, proctitis, cystitis, plexopathies, myelopathy, lymphoedema, burning perineum syndrome, and osteoradionecrosis [25].

Cervical cancer itself has been identified as a predictor of pain severity [32]. Despite its high burden, pain in women is often under-recognized and under-reported, which may underestimate its true prevalence and severity.

### Pelvic Floor Muscle Implications

The mechanisms underlying both urinary and fecal incontinence also involve pelvic floor muscle (PFM) dysfunction. Reductions in vaginal squeeze pressures, tonic contractions, anal maximal compression manometry, and maximal voluntary contraction in electromyography have been observed after RT [4, 8]. Fibrosis in the levator ani and urogenital diaphragm further impair sphincter closure for continence and for control of urgency episodes. RT also exerts late and progressive effects on PFM function, between 1 and 25 months after treatment [8].

### Limitations

The observational design of the studies included, preclude causal relationship analysis. In addition, the studies assessed patients at different time points after radiotherapy, which may lead to variability in reported symptoms. Also, the heterogeneity of studies (I^2^ > 80%) limits the definition of consistent patterns for some symptoms and requires cautious interpretation of the pooled prevalence estimates. This variability likely reflects differences in patient populations, cancer types and stages, radiotherapy parameters and techniques, timing of assessment, and symptom measurement tools. The wide temporal range of the studies included (1987–2023) may further introduce treatment era confounding, as treatment evolved over time. These factors underscore the need for greater standardization in symptom assessment and follow-up protocols to improve comparability across future studies. Moreover, the meta-regression findings should be interpreted with particular caution, as these analyses were based on a limited number of studies and may not have sufficient statistical power, making the identified associations exploratory rather than confirmatory.

### Strengths

This review has several notable strengths. First, to our knowledge, it is the first systematic review with meta-analysis specifically estimating the pooled prevalence of PFDs in women after pelvic radiotherapy, addressing a clinically relevant survivorship issue, given that improved survival after pelvic cancer means that more women are living with pelvic floor symptoms that substantially impact their quality of life.

Second, the study followed rigorous methodological standards, including protocol registration, a comprehensive multi-database search strategy, duplicate screening and data extraction, and risk of bias assessment using the JBI. Third, by applying meta-analysis and meta-regression, we were able not only to synthesize prevalence across diverse dysfunctions, but also to explore associations with clinically relevant moderators such as age, cancer stage, treatment modality, and follow-up time. Finally, the inclusion of a relatively large pooled sample (1667 participants) and the absence of publication bias strengthen the reliability of the estimates derived.

### Clinical Implications

Despite these limitations, our findings highlight the need to routinely inquire about pelvic floor symptoms during patient consultations, before and after treatment, throughout the follow-up.

Proactive screening, rather than waiting for patients’ spontaneous complaint reports can enable timely referrals to specialists. This approach directly addresses these distressing sequelae, moving beyond a sole focus on tumor control and recurrence, and ultimately has the potential to substantially improve patients’ quality of life. Pre-rehabilitation showed an effective protective effect, so this should also be suggested to the patients [2].

## Conclusion

Radiotherapy for pelvic malignancies is associated with urinary incontinence and overactive bladder symptoms. Studies were heterogeneous for bowel and sexual dysfunctions, but defecatory urgency significantly increased over time.

Routine assessment and proactive management of pelvic floor symptoms should be integrated into survivorship care. Interdisciplinary approaches, including physiotherapy, urogynecology, coloproctology, sexual health, and psycho-oncology, are essential to address the multifactorial nature of these conditions.

By shifting focus from tumor control alone to comprehensive survivorship care, clinicians can improve long-term quality of life for women treated with pelvic radiotherapy.

## Supplementary Information

Below are the links to the electronic supplementary material.

Supplementary file1 (PDF 177 KB)

Supplementary file2 (PDF 176 KB)

## Data Availability

All data relevant to this study are included in the article and its supplementary material as file 2.

## References

[1] Sung H, Ferlay J, Siegel RL, Laversanne M, Soerjomataram I, Jemal A, et al. Global Cancer Statistics 2020: GLOBOCAN estimates of incidence and mortality worldwide for 36 cancers in 185 countries. CA Cancer J Clin. 2021;71(3):209–49. 10.3322/caac.21660. 33538338

[2] Cai L, Wu Y, Xu X, Cao J, Li D. Pelvic floor dysfunction in gynecologic cancer survivors. Eur J Obstet Gynecol Reprod Biol. 2023;288:108–13. 10.1016/j.ejogrb.2023.07.010. 37499277

[3] Hsu WC, Chung NN, Chen YC, Ting LL, Wang PM, Hsieh PC, et al. Comparison of surgery or radiotherapy on complications and quality of life in patients with the stage IB and IIA uterine cervical cancer. Gynecol Oncol. 2009;115(1):41–5. 10.1016/j.ygyno.2009.06.028. 19615724

[4] Miguel TP, Laurienzo CE, Faria EF, Sarri AJ, Castro IQ, Júnior RJA, et al. Chemoradiation for cervical cancer treatment portends high risk of pelvic floor dysfunction. PLoS ONE. 2020;15(6):e0234389. 10.1371/journal.pone.0234389. PMC7292417 32530941

[5] Ye AL, Johnston E, Hwang S. Pelvic floor therapy and initial interventions for pelvic floor dysfunction in gynecologic malignancies. Curr Oncol Rep. 2024;26:212–20. 10.1007/s11912-024-01498-6. 38294706

[6] Cui YY, Wen LY, Chen XY, Bai XH. Pelvic floor dysfunction and its influencing factors during radiotherapy in cervical cancer survivors: a cross-sectional study. Eur J Oncol Nurs. 2023;64:102307. 10.1016/j.ejon.2023.102307. 37141663

[7] Shan X, Qian M, Wang L, Liu X. Prevalence of pelvic floor dysfunction and sexual dysfunction in cervical cancer survivors: a systematic review and meta-analysis. Int Urogynecol J. 2023;34(3):655–64. 10.1007/s00192-022-05326-y. 36001098

[8] Bernard S, Ouellet MP, Moffet H, Roy JS, Dumoulin C. Effects of radiation therapy on the structure and function of the pelvic floor muscles of patients with cancer in the pelvic area: a systematic review. J Cancer Surviv. 2016;10(2):351–62. 10.1007/s11764-015-0481-8. 26314412

[9] Page MJ, McKenzie JE, Bossuyt PM, Boutron I, Hoffmann TC, Mulrow CD, et al. The PRISMA 2020 statement: an updated guideline for reporting systematic reviews. Syst Rev. 2021;10(1):89. 10.1186/s13643-021-01626-4. PMC8008539 33781348

[10] Hazewinkel MH, Sprangers MAG, Velden J, Burger MPM, Roovers JPWR. Severe pelvic floor symptoms after cervical cancer treatment are predominantly associated with mental and physical well-being and body image. Int J Gynecol Cancer. 2012;22(1):154–60. 10.1097/IGC.0b013e3182332df8. 22080883

[11] Zhu L, Li X, Zhou C, Tong Y, Liu Z, Huang C. Pelvic floor dysfunction after colorectal cancer treatment is related to physical and psychological health and body image: a cross-sectional study. Eur J Oncol Nurs. 2023;67:102425. 10.1016/j.ejon.2023.102425. 37871415

[12] Abayomi J, Kirwan J, Hackett A. The prevalence of chronic radiation enteritis following radiotherapy for cervical or endometrial cancer and its impact on quality of life. Eur J Oncol Nurs. 2009;13(4):262–7. 10.1016/j.ejon.2009.02.007. 19640788

[13] Dunberger G, Lind H, Steineck G, Waldenström AC, Nyberg T, Al-Abany M, et al. Self-reported symptoms of faecal incontinence among long-term gynaecological cancer survivors and population-based controls. Eur J Cancer. 2010;46(3):606–15. 10.1016/j.ejca.2009.10.023. 19926277

[14] Hazewinkel MH, Sprangers MA, van der Velden J, van der Vaart CH, Stalpers LJ, Burger MP, et al. Long-term cervical cancer survivors suffer from pelvic floor symptoms: a cross-sectional matched cohort study. Gynecol Oncol. 2010;117(2):281–6. 10.1016/j.ygyno.2010.01.034. 20170944

[15] Kirchheiner K, Nout RA, Tanderup K, Lindegaard JC, Westerveld H, Haie-Meder C, et al. Manifestation pattern of early–late vaginal morbidity after definitive radiation (chemo)therapy and image-guided adaptive brachytherapy for locally advanced cervical cancer: an analysis from the EMBRACE study. Int J Radiat Oncol Biol Phys. 2014;89(1):88–95. 10.1016/j.ijrobp.2014.01.032. 24725693

[16] Lind H, Waldenström AC, Dunberger G, Al-Abany M, Alevronta E, Johansson KA, et al. Late symptoms in long-term gynaecological cancer survivors after radiation therapy: a population-based cohort study. Br J Cancer. 2011;105(6):737–45. 10.1038/bjc.2011.315. PMC3171018 21847122

[17] Parkin DE, Davis JA, Symonds RP. Long-term bladder symptomatology following radiotherapy for cervical carcinoma. Radiother Oncol. 1987;9(3):195–9. 10.1016/s0167-8140(87)80230-x. 3628856

[18] Vistad I, Cvancarova M, Fosså SD, Kristensen GB. Postradiotherapy morbidity in long-term survivors after locally advanced cervical cancer: how well do physicians’ assessments agree with those of their patients? Int J Radiat Oncol Biol Phys. 2008;71(5):1335–42. 10.1016/j.ijrobp.2007.12.030. 18355976

[19] Georg P, Boni A, Ghabuous A, Goldner G, Schmid MP, Georg D, et al. Time course of late rectal- and urinary bladder side effects after MRI-guided adaptive brachytherapy for cervical cancer. Strahlenther Onkol. 2013;189(7):535–40. 10.1007/s00066-013-0365-7. 23703404

[20] Noronha AF, de Mello Figueiredo E, Franco TM, Cândido EB, Silva-Filho AL. Treatments for invasive carcinoma of the cervix: what are their impacts on the pelvic floor functions? Int Braz J Urol. 2013;39(1):46–54. 10.1590/S1677-5538.IBJU.2013.01.07. 23489516

[21] Zomkowski K, Toryi AM, Sacomori C, Dias M, Sperandio FF. Sexual function and quality of life in gynecological cancer pre- and post-short-term brachytherapy: a prospective study. Arch Gynecol Obstet. 2016;294(4):833–40. 10.1007/s00404-016-4099-5. 27117556

[22] Peters M, Godfrey C, Mcinerney P, Soares C, Hanan K, Parker D. The Joanna Briggs Institute Reviewers’ Manual 2015: Methodology for JBI. Scoping Reviews. The Joanna Briggs Institute. Usp.br. 2015. Available from: https://repositorio.usp.br/item/002775594. Aug 2024

[23] Rostami P, Mostafaei H, Salehi-Pourmehr H, Hajebrahimi S. An updated systematic review and meta-analysis on the prevalence of female urinary incontinence in developing countries—a collaborative report by the International Continence Society (Developing World Committee) and the Iranian Research Center for Evidence-Based Medicine. Neurourol Urodyn. 2025;44(8):1639–68. 10.1002/nau.70127. 40836396

[24] Zhang L, Cai N, Mo L, Tian X, Liu H, Yu B. Global prevalence of overactive bladder: a systematic review and meta-analysis. Int Urogynecol J. 2025;36(8):1547–66. 10.1007/s00192-024-06029-2. PMC12464077 39951109

[25] Natuhwera G, Ellis P. The impact of chronic pelvic pain and bowel morbidity on quality of life in cervical cancer patients treated with radio (chemo) therapy. A systematic literature review. J Pain Res. 2025;18:597–618. 10.2147/JPR.S501378. PMC11809231 39931424

[26] Allafi AH, Al-Johani AS, Babukur RM, Fikri J, Alanazi RR, Ali SDMH, et al. The link between menopause and urinary incontinence: a systematic review. Cureus. 2024;16(10):e71260. 10.7759/cureus.71260. PMC11550778 39525118

[27] Yang X, Wang X, Gao Z, Li L, Lin H, Wang H, et al. The anatomical pathogenesis of stress urinary incontinence in women. Medicina Kaunas. 2022;59(1):5. 10.3390/medicina59010005. PMC9865065 36676629

[28] Yu Z, Xu C, Song B, Zhang S, Chen C, Li C. Tissue fibrosis induced by radiotherapy: current understanding of the molecular mechanisms, diagnosis and therapeutic advances. J Transl Med. 2023;21(1):708. 10.1186/s12967-023-04554-0. PMC10563272 37814303

[29] Jonker JE, Trzpis M, Broens PMA. Fecal continence for solid and liquid stool: the function of the anal–external sphincter continence reflex and the puborectal continence reflex. Dis Colon Rectum. 2020;63(10):1419–26. 10.1097/DCR.0000000000001615. 32969885

[30] Yeoh E, Sun WM, Russo A, Ibanez L, Horowitz M. A retrospective study of the effects of pelvic irradiation for gynecological cancer on anorectal function. Int J Radiat Oncol Biol Phys. 1996;35(5):1003–10. 10.1016/0360-3016(96)00147-2. 8751409

[31] Jensen PT, Froeding LP. Pelvic radiotherapy and sexual function in women. Transl Androl Urol. 2015;4(2):186–205. 10.3978/j.issn.2223-4683.2015.04.06. PMC4708128 26816824

[32] Donovan KA, Boyington AR, Judson PL, Wyman JF. Bladder and bowel symptoms in cervical and endometrial cancer survivors. Psychooncology. 2014;23(6):672–8. 10.1002/pon.3461. PMC4043946 24481859

