# Supplementary material for: Prevalence of Pelvic Floor Dysfunction in Women After Pelvic Radiotherapy: Systematic Review and Meta-Analysis
Source: Int Urogynecol J. 2026 Mar 13;37(9):2755–63. doi: 10.1007/s00192-026-06557-z (PMC13649904; doi:10.1007/s00192-026-06557-z)
Supplement: Supplementary file 2 [file 192_2026_6557_MOESM2_ESM.pdf]

Search Strategy

**PUBMED**	(((((Women[MeSH Terms]) OR (Women[Title/Abstract] OR Girls[Title/Abstract] OR Girl[Title/Abstract] OR Woman[Title/Abstract] OR "Women's Groups"[Title/Abstract] OR "Women Groups"[Title/Abstract] OR "Women's Group"[Title/Abstract])) OR (Female[MeSH Terms])) OR (Female[Title/Abstract] OR Females[Title/Abstract])) AND (((Pelvic Floor Disorders[MeSH Terms]) OR ("Pelvic Floor Disorders"[Title/Abstract] OR "Pelvic Floor Disorder"[Title/Abstract] OR "Pelvic Floor Diseases"[Title/Abstract] OR "Pelvic Floor Disease"[Title/Abstract])) OR ("dysfunctional pelvic floor"[Title/Abstract] OR "pelvic floor disfunction"[Title/Abstract] OR "pelvic floor dysfunction"[Title/Abstract] OR "pelvic wall disease"[Title/Abstract]))) AND ((((((((Radiotherapy[MeSH Terms]) OR (Radiotherapy[Title/Abstract] OR Radiotherapies[Title/Abstract] OR "Radiation Therapy"[Title/Abstract] OR "Radiation Therapies"[Title/Abstract] OR "Radiation Treatment"[Title/Abstract] OR "Radiation Treatments"[Title/Abstract] OR "Targeted Radiotherapies"[Title/Abstract] OR "Targeted Radiotherapy"[Title/Abstract] OR "Targeted Radiation Therapy"[Title/Abstract] OR "Targeted Radiation Therapies"[Title/Abstract])) OR ("radiation therapy"[Title/Abstract] OR "radiation treatment"[Title/Abstract] OR "radio therapy"[Title/Abstract] OR "irradiation therapy"[Title/Abstract] OR "irradiation treatment"[Title/Abstract] OR "fractionated radiotherapy"[Title/Abstract] OR "therapeutic radiology"[Title/Abstract])) OR ((Chemoradiotherapy[MeSH Terms]) OR (Chemoradiotherapy[Title/Abstract] OR Chemoradiotherapies[Title/Abstract] OR Radiochemotherapy[Title/Abstract] OR Radiochemotherapies[Title/Abstract] OR "Concurrent Chemoradiotherapy"[Title/Abstract] OR "Concurrent Chemoradiotherapies"[Title/Abstract] OR "Synchronous Chemoradiotherapy"[Title/Abstract] OR "Concurrent Radiochemotherapy"[Title/Abstract] OR "Concomitant Chemoradiotherapy"[Title/Abstract] OR "Concomitant Radiochemotherapy"[Title/Abstract]))) OR (Chemoradiation[Title/Abstract] OR radiochemotherapy[Title/Abstract])) OR ((Radiotherapy, Adjuvant[MeSH Terms]) OR ("Radiotherapy, Adjuvant"[Title/Abstract] OR "Adjuvant Radiotherapy"[Title/Abstract] OR "Adjuvant Radiotherapies"[Title/Abstract]))) OR ((Brachytherapy[MeSH Terms]) OR (Brachytherapy[Title/Abstract] OR "Radioisotope Brachytherapy"[Title/Abstract] OR Curietherapy[Title/Abstract] OR "Radioisotope Plaque Therapy"[Title/Abstract] OR "Surface Radiotherapy"[Title/Abstract] OR "Intracavity Radiotherapy"[Title/Abstract] OR "Interstitial Radiotherapy"[Title/Abstract] OR "Implant Radiotherapy"[Title/Abstract]))) AND (((((Pelvis[MeSH Terms]) OR (Pelvis[Title/Abstract] OR "Pelvic Region"[Title/Abstract])) OR (pelvic[Title/Abstract])) OR ((Pelvic Floor[MeSH Terms]) OR ("Pelvic Floor"[Title/Abstract] OR "Pelvic Diaphragm"[Title/Abstract]))) OR ("diaphragma pélvis"[Title/Abstract] OR "pelvis diaphragm"[Title/Abstract] OR "pelvis floor"[Title/Abstract])))
**PUBMED**<br>**PMC**	(((((Women[MeSH Terms]) OR (Women[Title/Abstract] OR Girls[Title/Abstract] OR Girl[Title/Abstract] OR Woman[Title/Abstract] OR "Women's Groups"[Title/Abstract] OR "Women Groups"[Title/Abstract] OR "Women's Group"[Title/Abstract])) OR (Female[MeSH Terms])) OR (Female[Title/Abstract] OR Females[Title/Abstract])) AND (((Pelvic Floor Disorders[MeSH Terms]) OR ("Pelvic Floor Disorders"[Title/Abstract] OR "Pelvic Floor Disorder"[Title/Abstract] OR "Pelvic Floor Diseases"[Title/Abstract] OR "Pelvic Floor Disease"[Title/Abstract])) OR ("dysfunctional pelvic floor"[Title/Abstract] OR "pelvic floor disfunction"[Title/Abstract] OR "pelvic floor dysfunction"[Title/Abstract] OR "pelvic wall disease"[Title/Abstract]))) AND ((((((((Radiotherapy[MeSH Terms]) OR (Radiotherapy[Title/Abstract] OR Radiotherapies[Title/Abstract] OR "Radiation Therapy"[Title/Abstract] OR "Radiation Therapies"[Title/Abstract] OR "Radiation Treatment"[Title/Abstract] OR "Radiation Treatments"[Title/Abstract] OR "Targeted Radiotherapies"[Title/Abstract] OR "Targeted Radiotherapy"[Title/Abstract] OR "Targeted Radiation Therapy"[Title/Abstract] OR "Targeted Radiation Therapies"[Title/Abstract])) OR ("radiation therapy"[Title/Abstract] OR "radiation treatment"[Title/Abstract] OR "radio therapy"[Title/Abstract] OR "irradiation therapy"[Title/Abstract] OR "irradiation treatment"[Title/Abstract] OR "fractionated radiotherapy"[Title/Abstract] OR "therapeutic radiology"[Title/Abstract])) OR ((Chemoradiotherapy[MeSH Terms]) OR (Chemoradiotherapy[Title/Abstract] OR Chemoradiotherapies[Title/Abstract] OR Radiochemotherapy[Title/Abstract] OR Radiochemotherapies[Title/Abstract] OR "Concurrent Chemoradiotherapy"[Title/Abstract] OR "Concurrent Chemoradiotherapies"[Title/Abstract] OR "Synchronous Chemoradiotherapy"[Title/Abstract] OR "Concurrent Radiochemotherapy"[Title/Abstract] OR "Concomitant Chemoradiotherapy"[Title/Abstract] OR "Concomitant Radiochemotherapy"[Title/Abstract]))) OR (Chemoradiation[Title/Abstract] OR

	radiochemotherapy[Title/Abstract])) OR ((Radiotherapy, Adjuvant[MeSH Terms]) OR ("Radiotherapy, Adjuvant"[Title/Abstract] OR "Adjuvant Radiotherapy"[Title/Abstract] OR "Adjuvant Radiotherapies"[Title/Abstract]))) OR ((Brachytherapy[MeSH Terms]) OR (Brachytherapy[Title/Abstract] OR "Radioisotope Brachytherapy"[Title/Abstract] OR Curietherapy[Title/Abstract] OR "Radioisotope Plaque Therapy"[Title/Abstract] OR "Surface Radiotherapy"[Title/Abstract] OR "Intracavity Radiotherapy"[Title/Abstract] OR "Interstitial Radiotherapy"[Title/Abstract] OR "Implant Radiotherapy"[Title/Abstract]))) AND (((((Pelvis[MeSH Terms]) OR (Pelvis[Title/Abstract] OR "Pelvic Region"[Title/Abstract])) OR (pelvic[Title/Abstract])) OR ((Pelvic Floor[MeSH Terms]) OR ("Pelvic Floor"[Title/Abstract] OR "Pelvic Diaphragm"[Title/Abstract]))) OR ("diaphragma pélvis"[Title/Abstract] OR "pelvis diaphragm"[Title/Abstract] OR "pelvis floor"[Title/Abstract])))
BVS / BIREME<br>MEDLINE<br>IBECS<br>LILACS	((women OR girls OR girl OR woman OR "Women's Groups" OR "Women Groups" OR "Women's Group") OR (female OR females)) AND (("Pelvic Floor Disorders" OR "Pelvic Floor Disorder" OR "Pelvic Floor Diseases" OR "Pelvic Floor Disease") OR ("dysfunctional pelvic floor" OR "pelvic floor disfunction" OR "pelvic floor dysfunction" OR "pelvic wall disease")) AND ((radiotherapy OR radiotherapies OR "Radiation Therapy" OR "Radiation Therapies" OR "Radiation Treatment" OR "Radiation Treatments" OR "Targeted Radiotherapies" OR "Targeted Radiotherapy" OR "Targeted Radiation Therapy" OR "Targeted Radiation Therapies") OR ("radiation therapy" OR "radiation treatment" OR "radio therapy" OR "irradiation therapy" OR "irradiation treatment" OR "fractionated radiotherapy" OR "therapeutic radiology") OR (chemoradiotherapy OR chemoradiotherapies OR radiochemotherapy OR radiochemotherapies OR "Concurrent Chemoradiotherapy" OR "Concurrent Chemoradiotherapies" OR "Synchronous Chemoradiotherapy" OR "Concurrent Radiochemotherapy" OR "Concomitant Chemoradiotherapy" OR "Concomitant Radiochemotherapy") OR (chemoradiation OR radiochemotherapy) OR ("Radiotherapy, Adjuvant" OR "Adjuvant Radiotherapy" OR "Adjuvant Radiotherapies") OR (brachytherapy OR "Radioisotope Brachytherapy" OR curietherapy OR "Radioisotope Plaque Therapy" OR "Surface Radiotherapy" OR "Intracavity Radiotherapy" OR "Interstitial Radiotherapy" OR "Implant Radiotherapy")) AND ((pelvis OR "Pelvic Region") OR (pelvic) OR ("Pelvic Floor" OR "Pelvic Diaphragm") OR ("diaphragma pélvis" OR "pelvis diaphragm" OR "pelvis floor"))
**SCOPUS**	((TITLE-ABS-KEY (women OR girls OR girl OR woman OR "Women's Groups" OR "Women Groups" OR "Women's Group") OR TITLE-ABS-KEY (female OR females))) AND ((TITLE-ABS-KEY ("Pelvic Floor Disorders" OR "Pelvic Floor Disorder" OR "Pelvic Floor Diseases" OR "Pelvic Floor Disease") OR TITLE-ABS-KEY ("dysfunctional pelvic floor" OR "pelvic floor disfunction" OR "pelvic floor dysfunction" OR "pelvic wall disease"))) AND ((TITLE-ABS-KEY (radiotherapy OR radiotherapies OR "Radiation Therapy" OR "Radiation Therapies" OR "Radiation Treatment" OR "Radiation Treatments" OR "Targeted Radiotherapies" OR "Targeted Radiotherapy" OR "Targeted Radiation Therapy" OR "Targeted Radiation Therapies") OR TITLE-ABS-KEY ("radiation therapy" OR "radiation treatment" OR "radio therapy" OR "irradiation therapy" OR "irradiation treatment" OR "fractionated radiotherapy" OR "therapeutic radiology") OR TITLE-ABS-KEY (chemoradiotherapy OR chemoradiotherapies OR radiochemotherapy OR radiochemotherapies OR "Concurrent Chemoradiotherapy" OR "Concurrent Chemoradiotherapies" OR "Synchronous Chemoradiotherapy" OR "Concurrent Radiochemotherapy" OR "Concomitant Chemoradiotherapy" OR "Concomitant Radiochemotherapy") OR TITLE-ABS-KEY (chemoradiation OR radiochemotherapy) OR TITLE-ABS-KEY ("Radiotherapy, Adjuvant" OR "Adjuvant Radiotherapy" OR "Adjuvant Radiotherapies") OR TITLE-ABS-KEY (brachytherapy OR "Radioisotope Brachytherapy" OR curietherapy OR "Radioisotope Plaque Therapy" OR "Surface Radiotherapy" OR "Intracavity Radiotherapy" OR "Interstitial Radiotherapy" OR "Implant Radiotherapy"))) AND ((TITLE-ABS-KEY (pelvis OR "Pelvic Region") OR TITLE-ABS-KEY (pelvic) OR TITLE-ABS-KEY ("Pelvic Floor" OR "Pelvic Diaphragm") OR TITLE-ABS-KEY ("diaphragma pélvis" OR "pelvis diaphragm" OR "pelvis floor")))
**WEB OF SCIENCE**	Women OR Girls OR Girl OR Woman OR "Women's Groups" OR "Women Groups" OR "Women's Group" (Topic) or Female OR Females (Topic) and Preprint Citation Index (Exclude – Database) AND "Pelvic Floor Disorders" OR "Pelvic Floor Disorder" OR "Pelvic Floor Diseases" OR "Pelvic Floor Disease" (Topic) or "dysfunctional pelvic floor" OR "pelvic floor disfunction" OR "pelvic floor dysfunction" OR "pelvic wall disease" (Topic) and Preprint Citation Index (Exclude – Database) AND Radiotherapy OR Radiotherapies OR "Radiation Therapy" OR "Radiation Therapies" OR "Radiation Treatment" OR "Radiation Treatments" OR "Targeted Radiotherapies" OR "Targeted Radiotherapy" OR "Targeted Radiation Therapy" OR "Targeted Radiation Therapies" (Topic) or "radiation therapy" OR "radiation treatment" OR "radio therapy" OR "irradiation therapy" OR "irradiation treatment" OR "fractionated radiotherapy" OR

	"therapeutic radiology" (Topic) or Chemoradiotherapy OR Chemoradiotherapies OR Radiochemotherapy OR Radiochemotherapies OR "Concurrent Chemoradiotherapy" OR "Concurrent Chemoradiotherapies" OR "Synchronous Chemoradiotherapy" OR "Concurrent Radiochemotherapy" OR "Concomitant Chemoradiotherapy" OR "Concomitant Radiochemotherapy" (Topic) or Chemoradiation OR radiochemotherapy (Topic) or "Radiotherapy, Adjuvant" OR "Adjuvant Radiotherapy" OR "Adjuvant Radiotherapies" (Topic) or Brachytherapy OR "Radioisotope Brachytherapy" OR Curietherapy OR "Radioisotope Plaque Therapy" OR "Surface Radiotherapy" OR "Intracavity Radiotherapy" OR "Interstitial Radiotherapy" OR "Implant Radiotherapy" (Topic) and Preprint Citation Index (Exclude – Database) AND Pelvis OR "Pelvic Region" (Topic) or pelvic (Topic) or "Pelvic Floor" OR "Pelvic Diaphragm" (Topic) or "diaphragma pélvis" OR "pelvis diaphragm" OR "pelvis floor" (Topic) and Preprint Citation Index (Exclude – Database)<br><br>https://www.webofscience.com/wos/alldb/summary/7e2738ae-c093-4a2c-a465-201078a6faa5-01005d5072/relevance/1
**EMBASE**	('female'/syn OR girls:ti,ab,kw OR girl:ti,ab,kw OR 'womens groups':ti,ab,kw OR 'women groups':ti,ab,kw OR 'womens group':ti,ab,kw) AND 'pelvic floor disorder'/syn AND ('radiotherapy'/syn OR 'chemoradiotherapy'/syn OR 'adjuvant radiotherapy'/syn OR 'brachytherapy'/syn) AND ('pelvis'/syn OR 'pelvic floor'/syn) AND ([embase]/lim NOT ([embase]/lim AND [medline]/lim) OR ([medline]/lim NOT ([embase]/lim AND [medline]/lim) NOT ([embase classic]/lim AND [medline]/lim)))
**COCHRANE LIBRARY**	"diaphragma pélvis" OR "pelvis diaphragm" OR "pelvis floor" OR (Women OR Girls OR Girl OR Woman OR "Women's Groups" OR "Women Groups" OR "Women's Group"):ti,ab,kw OR MeSH descriptor: [Female] in all MeSH products OR (Female OR Females):ti,ab,kw AND MeSH descriptor: [Pelvic Floor Disorders] explode all tree OR ("Pelvic Floor Disorders" OR "Pelvic Floor Disorder" OR "Pelvic Floor Diseases" OR "Pelvic Floor Disease"):ti,ab,kw OR ("dysfunctional pelvic floor" OR "pelvic floor disfunction" OR "pelvic floor dysfunction" OR "pelvic wall disease"):ti,ab,kw AND MeSH descriptor: [Radiotherapy] explode all trees OR (Radiotherapy OR Radiotherapies OR "Radiation Therapy" OR "Radiation Therapies" OR "Radiation Treatment" OR "Radiation Treatments" OR "Targeted Radiotherapies" OR "Targeted Radiotherapy" OR "Targeted Radiation Therapy" OR "Targeted Radiation Therapies"):ti,ab,kw OR ("radiation therapy" OR "radiation treatment" OR "radio therapy" OR "irradiation therapy" OR "irradiation treatment" OR "fractionated radiotherapy" OR "therapeutic radiology"):ti,ab,kw OR MeSH descriptor: [Chemoradiotherapy] explode all trees OR (Chemoradiotherapy OR Chemoradiotherapies OR Radiochemotherapy OR Radiochemotherapies OR "Concurrent Chemoradiotherapy" OR "Concurrent Chemoradiotherapies" OR "Synchronous Chemoradiotherapy" OR "Concurrent Radiochemotherapy" OR "Concomitant Chemoradiotherapy" OR "Concomitant Radiochemotherapy"):ti,ab,kw OR (Chemoradiation OR radiochemotherapy):ti,ab,kw OR MeSH descriptor: [Radiotherapy, Adjuvant] explode all trees OR ("Radiotherapy, Adjuvant" OR "Adjuvant Radiotherapy" OR "Adjuvant Radiotherapies"):ti,ab,kw OR MeSH descriptor: [Brachytherapy] explode all trees OR (Brachytherapy OR "Radioisotope Brachytherapy" OR Curietherapy OR "Radioisotope Plaque Therapy" OR "Surface Radiotherapy" OR "Intracavity Radiotherapy" OR "Interstitial Radiotherapy" OR "Implant

		Radiotherapy"):ti,ab,kw AND MeSH descriptor: [Pelvis] explode all trees OR (Pelvis OR "Pelvic Region"):ti,ab,kw OR (pelvic):ti,ab,kw OR MeSH descriptor: [Pelvic Floor] explode all trees OR "Pelvic Floor" OR "Pelvic Diaphragm"):ti,ab,kw OR ("diaphragma pélvis" OR "pelvis diaphragm" OR "pelvis floor"):ti,ab,kw	
